# The Applications of Enamel Matrix Derivative in Implant Dentistry: A Narrative Review

**DOI:** 10.3390/ma14113045

**Published:** 2021-06-03

**Authors:** Alice Alberti, Luca Francetti, Silvio Taschieri, Stefano Corbella

**Affiliations:** 1 Department of Biomedical, Surgical and Dental Sciences, Università degli Studi di Milano, 20161 Milan, Italy; alice.alberti@unimi.it (A.A.); luca.francetti@unimi.it (L.F.); silvio.taschieri@unimi.it (S.T.); 2 IRCCS Istituto Ortopedico Galeazzi, 20161 Milan, Italy; 3 Department of Oral Surgery, Institute of Dentistry, I. M. Sechenov First Moscow State Medical University, 119435 Moscow, Russia

**Keywords:** amelogenin, bone regeneration, dental implants, enamel matrix derivative, peri-implantitis

## Abstract

Enamel matrix derivative (EMD) has been successfully used for periodontal regeneration in intrabony defects. Recently, its use for peri-implant bone regeneration has also been hypothesized. The aim of this paper is to review preclinical and clinical studies investigating the use of EMD in correspondence with titanium implants, alone or as an adjunct to other biomaterials. Clinical trials and case series with more than five cases were included. Seven in vitro studies evaluated the effect of EMD, placed on titanium surfaces: An increase in proliferation and viability of osteoblasts was observed in all but two studies. An increase in TGF-β1 and osteocalcin production, alkaline phosphatase activity, and angiogenesis was also reported. Nine animal studies investigated the use of EMD at implant placement or for bone regeneration of peri-implant bone defects, and some of them reported a significant increase in bone formation or bone-to-implant contact. In four of eleven clinical trials on humans, EMD was successfully used at implant placement. The other seven evaluated the use of EMD in protocols for the treatment of peri-implantitis. In conclusion, the results of EMD seem promising, but further randomized clinical trials are needed to evaluate its efficacy.

## 1. Introduction

Enamel matrix derivative (EMD) is a purified acid extract of proteins extracted from porcine enamel, that were introduced after a long period of testing as a biological medium for enhancing periodontal tissue regeneration [1,2]. The major component (more than 95%) of EMD is represented by amelogenins, a family of hydrophobic proteins that constitute the unique active component of the product [3]. Amelogenins are involved in a number of biological functions that are related to the regulation of fibroblasts and osteoblasts, enhancing their activation and, ultimately, their activity [3]. Such proteins are normally present during the development of the attachment apparatus of the tooth and have been proven to promote regenerative responses in the periodontal ligament. The mechanisms have not been fully explained, but it is known that amelogenins, when applied to a conditioned root surface, precipitate to form an insoluble extracellular matrix with high affinity for hydroxyapatite and collagen, which can interact with the surrounding cells and thus initiates the periodontal regeneration [3].

The use of EMD was widely validated in periodontal surgery by a number of systematic reviews of the literature [4,5,6,7]. The effect of such biomaterial was demonstrated for the regeneration of periodontal intrabony defects, alone or as an adjunct to bone substitute material, even when using mini-invasive surgical approaches [6]. Indeed, in one recent systematic review of the literature EMD performed better than platelet derivatives and better than the bone substitute alone in regenerative/reconstructive procedures [6].

Interestingly, a growing evidence supported the use of EMD in the surgical treatment of gingival recessions, associated or not with connective tissue graft [4]. The systematic review published in 2021 on the use of EMD to treat Miller class I or II gingival recessions proved the beneficial effect of EMD application for all the techniques tested [4].

Since the effects of amelogenins on cells involved in periodontal regeneration found substantial support in the literature, the hypothesis that EMD could stimulate osteoblast and bone regeneration has been studied both in recently published animal [8,9] and human studies [10,11]. Jung et al. [9] compared the effect of EMD-liquid as an adjunct to biphasic calcium phosphate (BCP) in a rabbit calvarial model, as evaluated through micro-CT and histomorphometric analysis. When EMD was applied, new bone formation was significantly higher and the material area was significantly lower, indicating accelerated graft degradation. A higher amount of newly formed bone at 6 months was also observed in a human RCT investigating the effect of EMD in association with deproteinized bovine bone mineral (DBBM) as compared to DBBM alone for maxillary sinus floor elevation [10]. The same results were obtained at 4 months in another RCT testing the same biomaterials for ridge preservation after maxillary anterior teeth extraction [11]. A recent in vitro study [12] investigated the effect of EMD on human osteoclasts precursors after interaction with activated endothelium, finding a concentration-dependent inhibition of osteoclastogenesis. This suggests that EMD could affect bone resorption, thus promoting bone regeneration during periodontal therapy.

The aim of the present study was to present a narrative review of the scientific literature about the use of EMD in correspondence of titanium implants, also evaluating laboratory studies with methods compatible with the purpose of the research.

## 2. Materials and Methods

Although the present is a narrative review of the literature, we aimed at performing a thorough review of the existing literature by combining appropriate keywords (MeSH terms/emtree terms and free text strings) (Appendix A) in order to identify all the studies published about the behavior, in preclinical and clinical studies, of enamel matrix derivative alone or as an adjunct to other biomaterials when used in association with dental implants. Moreover, we searched for studies investigating the application of enamel matrix derivative for the treatment of peri-implant inflammatory diseases (peri-implant mucositis and peri-implantitis).

We decided to include laboratory in vitro studies, animal studies, and clinical studies on humans.

The primary outcome we would like to assess was the efficacy in the experimental settings of their use, as compared with other treatment options, in similar clinical conditions. As for clinical studies, randomized controlled clinical trials, prospective and retrospective studies with an adequate study design were included. Case reports and case series with less than five cases were excluded.

Due to the relatively low number and the heterogeneity of the design of studies dealing with the predetermined focus of the present review we decided to present the results of the studies included in a narrative and critical manner, avoiding performing a meta-analysis or quantitative synthesis.

The authors reported the results of the studies separately depending on the type of the study included (in vitro, animal studies, and clinical studies on humans). For each study, the following parameters were extracted and considered: Name of the authors and year of publication, tested cells (in vitro study), characteristics of the implant, animal model (in animal studies) surface, characteristics of the population, methods and synthesis of the results obtained.

## 3. Results

The search we performed resulted in 27 papers to be considered, that are described analytically in the following paragraphs.

### 3.1. In Vitro Studies

Seven in vitro studies observed the behavior of enamel matrix derivatives on titanium surface, evaluating the ability of inducing and promoting osteoprogenitor cells [13,14,15,16,17], primary gingival fibroblasts [18] or endothelial cells [19] (Table 1).

The study by Schwarz et al. published in 2004 investigated the effects of EMD on human osteoblasts-like cells attachment, viability, and proliferation on titanium implants with sand-blasted acid-etched surfaces (SLA), evaluating the samples for 6 days [13]. The authors found that cell proliferation and viability were improved by EMD exposure following a concentration-dependent pattern, having better outcomes after 6 days.

On the other hand, one study published in 2007, where rat osteoblasts were cultivated on either phosphate and non-phosphate titanium discs with or without the adjunct of EMD [14], did not reveal any significant advantage of one group over another one, even though it was observed that EMD could initiate an early TGF-β1 production, without any difference in the medium and long period.

In one similar study performed by Miron et al. in 2010, rat calvarial osteoblasts were cultured on titanium surfaces with or without EMD up to 4 weeks [15]. In the particular conditions of the research EMD proved to be effective in significantly augmenting cell spreading at 2 and 4 h and the proliferation of osteoblasts after 3 to 7 days, independently from the titanium surface substrate (smooth or SLA). The authors also registered an increase in alkaline phosphatase activity and in osteocalcin gene expression.

The same increase in alkaline phosphatase activity and osteocalcin production was found in one study by Qu et al. in 2011 [16], where MG-63 osteoblast-like cells were seeded on disks of SLA titanium and cultured for 7 days, and then they were ‘stimulated’ with EMD for 48 h. However, the authors reported a decrease in osteoblasts proliferation with high EMD concentrations within this period of observation.

The study by Shi et al. published in 2017 reported a similar decrease in cell proliferation on acid-etched and SLA surfaces after 48 h. Moreover, the authors presented data on the ability of EMD to stimulate angiogenic behavior on titanium disc surfaces (smooth, acid-etched or SLA) of cultivated endothelial cells [19]. Interestingly, EMD appeared to stimulate angiogenesis on SLA titanium.

Gingival fibroblasts proliferation on SLA and smooth surfaces coated with EMD was studied in the paper published in 2016 by Wang et al. [18]. The preparation of EMD was similar to what was presented in previous studies [15,16]. Cell adhesion, proliferation, and morphology was examined for up to 5 days after exposure. On all the surfaces examined, EMD was able to increase cell proliferation of gingival proliferation on SLA and smooth surfaces.

More recently, the study by Ramenzoni et al. published in 2020, aimed at evaluating the response of MC3T3 osteoblast-like cells cultivated on SLA titanium disks with EMD or dentin matrix derivative (DMD) or left uncoated [17]. Interestingly, EMD and DMD acted similarly in the experimental conditions, both increasing the osteoblasts proliferation and activity.

### 3.2. In Vivo Studies on Animal Models

Nine studies examine the behavior of EMD in the presence of titanium surfaces in animal models (Table 2) [20,21,22,23,24,25,26,27,28].

Three animal studies used evaluated the effect of EMD at implant placement through similar protocols [20,22,25]. The EMD application was evaluated in rats in the study by Shimizu-Ishiura, published in 2002, by wetting titanium mini-implants at the time of placement [20]. Rats were sacrificed after 4, 7, 14, and 30 days, finding that EMD was able to enhance trabecular bone formation around titanium implants after implantation.

No differences in bone formation between the test (implants wetted with EMD at the time of implant placement) and negative control group were found in the study published in 2003 by Franke Stenport and Johansson on 36 commercially pure titanium implants placed in rabbits [22].

In an animal study performed by Birang et al. in 2012, 12 titanium implants were positioned in Iranian dogs, half of them wetted with EMD before insertion [25]. Bone formation was observed after 4 weeks from the surgery in both groups, but the osteoblasts activity was more pronounced in the EMD group.

The application of EMD in implant placement procedures was also part of the protocol called ‘socket-shield technique’ described by Hurzeler et al. in 2010, presenting the results of one study in one beagle dog [24]. In detail, implants were placed with or without contact with the radicular fragment and EMD was applied: Osseointegration occurred in all implants, together with the formation of new cementum for implants placed in direct contact with the radicular shield.

The paper by Casati et al. studied the histomorphometric results of EMD (with or without GBR) application for the treatment of peri-implant bone dehiscences in mongrel dogs, created ad hoc at the time of implant placement [21]. Bone-to-implant contact (BIC) was higher in test groups than in the negative control group, but without any statistically significant difference. Interestingly, no differences could be found between GBR alone and EMD alone, thus demonstrating a substantial bone healing stimulation with EMD.

Similarly, the study by Craig et al., published in 2006 in minipig studied EMD in conjunction with dental implants, in the presence of cultivated periodontal ligament and gingival connective tissues cells, for filling ad hoc created circumferential bone dehiscences [23]. The histomorphometric results found that the amount of bone-to-implant contact could be positively influenced by the presence of EMD.

The use of EMD in conjunction with bone substitutes such as deproteinized bovine bone mineral (DBBM) and biphasic calcium phosphate (BCP), in the presence of peri-implant bone dehiscences was studied in the research published in 2015 by Wen et al. [26]. Even though an effect of EMD was observed on bone regeneration, no significant differences could be observed as compared to the control groups.

BCP embedded with EMD was examined also in the study published by Lim et al. in 2016 [27]. In five mongrel dogs, peri-implant defects were created and filled with BCP alone, EMD alone or EMD and BCP, and bone regeneration was evaluated over time. No additional effect was observed for EMD and BCP-EMD groups, with regards to bone-to-implant contact and bone fill.

The ability of EMD to enhance bone regeneration when associated with DBBM for the treatment of vestibular peri-implant bone dehiscence in six beagles was studied recently by Ikawa et al., which found that EMD could improve significantly the augmented area and the bone-to-implant contact [28].

### 3.3. Clinical Studies on Humans

The search retrieved eleven papers on the use of EMD in the presence of dental implants in humans (Table 3) [29,30,31,32,33,34,35,36,37,38,39]. The studies reported the results both on soft and hard tissues.

Four studies evaluated the effect of EMD at implant placement [29,32,38,39]. One clinical report by Cangini and Cornelini, evaluated EMD as compared to the bioresorbable collagen membrane for enhancing bone and soft tissue healing around implants positioned immediately after extraction [29]. In 32 patients, half of the space between the implants and socket walls was filled with EMD and half covered with the bioresorbable membrane, and periodontal parameters were evaluated after 12 months, observing that the use of membrane performed better than EMD in this particular condition.

The effect of EMD on soft tissues after implant placement was the object of one clinical study published in 2015 on a total of five patients [32]. The histologic outcomes of biopsies retrieved 14 days after implant placement showed that EMD caused an increase of microvessel density as compared to control sites.

More recently, the research by Cardaropoli et al. was focused on the EMD application at the level of the hard and soft tissues, at the time of immediately loaded screwed restoration placement [38,39]. The soft tissues esthetics were satisfactory and stable after one year, together with the height and width of the bone crest evaluated through radiographs and CBCTs.

The other papers focused on the use of EMD within clinical protocols for the treatment of peri-implantitis, with a non-surgical [33,35] or surgical approach [30,31,34,36,37]. The case series by Froum et al. in 2012 described a protocol for the treatment of peri-implantitis that involved the use of EMD and presented the results of 3–7.5 years of such treatment on 51 affected implants [30]. Even though the results of such protocol appeared encouraging, the effects of EMD on bone regeneration could not be evaluated separately. The report was updated in 2015 (on 170 implants in 100 patients with 2–10 years follow-up) with similar results [31].

In the study by Faramarzi et al. published in 2015, EMD was compared to minocycline spheres as an adjunct to nonsurgical therapy for the treatment of peri-implant mucositis [33]. A total of 64 implants (divided into three groups) were treated and then followed-up for 3 months, evaluating clinical and microbiological outcomes. Both the use of EMD and minocycline resulted in better outcomes than the control group as an adjunct to the nonsurgical treatment of peri-implant mucositis. Another study of the same research group compared, with a similar protocol, EMD plus nonsurgical treatment and nonsurgical treatment alone in 46 patients with peri-implant mucositis [35]. After 3 months, the clinical and laboratory parameters improved significantly in the EMD group as compared to controls.

Isehed et al. published the results of one randomized controlled clinical trial on the use of EMD after surface debridement and decontaminations of implants presenting a bone dehiscence due to peri-implantitis [34,36]. The study was performed on 26 subjects and they were followed-up for up to 5 years. After 12 months, the EMD group showed increased marginal bone level than the control group and after 5 years it was confirmed that EMD was appositively associated with implant survival over time.

Moreover, the treatment of peri-implantitis by means of the adjunctive use of EMD was the objective of the study by Esberg et al. [37]. The protocol was the same as used by Isehed et al., namely open flap debridement and decontamination, with or without the adjunct of EMD before flap closure. It was demonstrated that EMD is related to implant survival over time, expressing a different proteomic expression profile.

## 4. Discussion

The present study aimed at understanding the efficacy and effectiveness of the use of EMD in correspondence of dental implants and peri-implant tissues in general.

The available scientific literature did not allow performing a meta-analysis, due to the large heterogeneity among the studies and to the low number of randomized clinical trials. However, we attempted to provide narratively the outcomes of the research in this particular field.

The laboratory studies included in the review were mainly about the effect of EMD on osteoblasts activity in general on titanium surfaces, in most studies SLA. The effect of EMD on cell proliferation was recognized in a substantial number of laboratory studies [40]. Indeed, it was observed that EMD in a liquid carrier could significantly improve osteoblasts and periodontal ligament cells growth and differentiation, with more expressions of genes codifying for collagen and osteocalcin [41]. Such stimulation was observed also towards the expression of connective tissue growth factors in periodontal ligament cells [42,43]. In general, the activity of EMD on the adhesion, proliferation, and activity of cells involved in bone regeneration was evaluated in addition to bone substitutes, such as DBBM, etc. [44,45,46]. With regards to the adsorption of EMD to the surfaces of the bone substitute material, a liquid carrier was demonstrated to be more efficient in the laboratory conditions, augmenting the surface of the grafting material coated by EMD [44]. In our review, the results of most laboratory studies confirmed that fibroblast and osteoblasts stimulation by the EMD application could be obtained also on titanium surfaces, with a significant effect as compared to controls, despite the fact that controversial results were obtained in two of the included studies [16,19].

Studies on animals are required to test the outcomes of bone grafting procedures performed using bone substitutes or other agents that aimed at increasing the regenerative potential [47].

Due to the heterogeneity in the materials and methods of the considered studies, it is not possible, in the present review, to find a substantial and proven effect of EMD on animal models on bone regeneration. It should be highlighted that EMD was safe, and its performances resulted at least non-inferior as compared to the control groups in two studies [20,21]. It is notable that in all animal studies, the defects were created *ad hoc*, and they were not of infective origin.

Several clinical studies reported the results of using EMD associated with dental implants placed in humans. As stated before, it was not possible to perform any quantitative synthesis of the results, and so the outcomes should be considered with caution, since in general the level of evidence was low.

In the studies included in the present review, EMD was tested for bone regeneration, for improving the soft tissue healing, and in protocol for the treatment of peri-implant mucositis and peri-implantitis. In general terms, EMD could improve bone-to-implant contact in immediately positioned implants even though the long-term effects on bone stability and on implant survival need to be confirmed by more studies.

The results of EMD were very promising, in the clinical studies included in the review, as an adjunct to other therapies for the treatment of peri-implant mucositis or peri-implantitis. The research performed by Froum et al., whose results were published in two papers, proposed the use of EMD in a complex surgical treatment protocol for peri-implantitis that included flap reflection, surface decontamination with chlorhexidine or a dedicated brush, platelet-derived growth factor, and guided bone regeneration with a bone substitute [30,31]. The results of the protocol proposed were similar to those presented in scientific literature after treatment of peri-implantitis and under an adequate protocol for supportive care [48]. Moreover, the promising results of the use of EMD were confirmed in one comparative clinical trial in which EMD was used associated with open flap debridement of implant surface, although the number of cases examined in the medium term (3–5 years) was relatively low [34,36,37]. Furthermore, the same positive results could be observed in the treatment of peri-implant mucositis, as an adjunct to standard mechanical therapy and biofilm removal, although there is no clear evidence of a superior effect of such protocol over the standard treatment [33,35]. As it was reported recently, it should be highlighted that nonsurgical therapy, without any additional chemical or mechanical agents, is sufficient for the resolution of peri-implant mucositis [49].

The stability and the anatomical characteristics of the so-called ‘implant supracrestal complex (ISC)’ are fundamental for maintaining oral hygiene and for limiting the possibility of occurring in peri-implant inflammatory diseases [50]. The EMD are adopted for increasing the stability and the health status of ISC in general, showing a substantial improvement in soft tissue healing but we have no information on the long-term clinical outcomes, since a number of factors could be more important in determining implant success over time.

Although the review found a moderate support in scientific literature on the use of EMD in the presence of dental implants, the results of the studies included should be considered with caution and the limitations of the present study have to be acknowledged. First, we had no possibility of performing any quantitative synthesis of the results, so the outcomes of each study could not be pooled together. Then, the studies included presented a substantial heterogeneity in study methods and in the protocols adopted, and this aspect does not allow proposing any standard of care. Moreover, animal studies presented a low evidence of the efficacy of EMD in laboratory conditions.

## 5. Conclusions

In conclusion, the present paper found a sparse evidence on the efficacy of the use of EMD for increasing bone regeneration and as an adjunct for the treatment of peri-implant diseases. However, in all studies, the EMD application was demonstrated as safe and not inferior to control protocols with regards to both clinical and laboratory outcomes. The promising, although limited, results might deserve to be studied further, in well-designed controlled clinical trials with a medium to long follow-up period and adequate sample size, in order to improve bone regeneration in particularly challenging conditions as peri-implant tissue.

## Figures and Tables

**Table 1:** In vitro studies that observed the behavior of EMD on titanium surface.

Authors	Year	Tested Cells	Titanium Surface	Methods	Outcomes and Observation Periods	Results
Schwarz et al. [13]	2004	Human osteoblast-like cells	SLA	Cells placed on titanium discs with medium alone or +EMD at different concentrations (25, 50, 100, and 200 μg/mL); the medium was changed after 3 days without EMD	Adhesion: 1, 3, and 6 days Proliferation, viability: 1, 3, and 6 days Cell morphology: 1, 3, and 6 days	Concentration-dependent increase in proliferation; statistically significant higher increase in viability with 100 and 200 μg/mL EMD at day 6 as compared to control
Dacy et al. [14]	2007	Rat osteoblasts	Phosphated and non-phosphated	Cells placed on titanium discs with medium alone or +EMD (180 μg); the medium was changed every 2 days for 28 days	Adhesion: every 2 days up to 28 days nodule formation and mineralization: every 2 days up to 28 days TGF-β1 and IL-1β production: every 2 days up to 28 days	Increased production of TGF-β1 for up to 8 days
Miron et al. [15]	2010	Rat calvarial osteoblasts	Smooth and SLA	Cells placed on titanium discs with medium alone or +EMD, cultured from 1 h to 4 weeks	Adhesion: 2, 4, and 8 h Cell spreading: 30 min, 2 and 4 h Proliferation: 1, 3, 5, and 7 days ALP activity: 1, 2, and 3 weeks Gene expression (Runx2, BSP, OC): 1, 7, 14, 21, and 28 days	Increased cell spreading after 2 and 4 h and increased proliferation after 3 to 7 days; increased ALP activity; increased levels of mRNA encoding bone sialoprotein and osteocalcin
Qu et al. [16]	2011	osteoblast-like MG-63 cells	SLA	Cells cultured on titanium discs for 7 days; the medium was changed every 2 days; EMD addition in test groups at different concentrations (12.5, 25, 50, and 100 μg/mL) at day 7	Proliferation, viability: 48 h ALP activity, OC production: 48 h Gene expression (OPG, RANKL): 48 h	Dose-dependent decrease in proliferation and viability at 50 and 100 μg/mL; increased ALP activity, osteocalcin production, and mRNA expression level of OPG at 50 and 100 μg/mL
Wang et al. [18]	2016	Human primary gingival fibroblasts	Smooth and SLA	Cells placed on titanium discs with medium alone or +EMD	Adhesion: 2, 4, and 8 h Proliferation: 1, 3, and 5 days Morphology: 2, 4, 8, and 24 h Gene expression (COL1A1, VEGF-A, FN1): 5 days Synthesis of collagen matrix (collagen type I staining): 7 and 14 days	Increased cell spreading and proliferation; increased mRNA levels of VEGF-A and fibronectin, increased extracellular matrix synthesis of collagen type I
Shi et al. [19]	2017	HUVECs	smooth, acid-etched or SLA	Cells cultured on titanium discs for 24 h; EMD addition (50 μg/mL) after 24 h	Proliferation, viability: 48 h Angiogenic activity (expression of genes ICAM-1, EPCR, E-selectin, vWF, Ang-2): 2 days	Decrease in proliferation/viability on acid-etched and SLA titanium; increased ICAM-1, EPCR, E-selectin, and vWF on SLA surface
Ramenzoni et al. [17]	2020	MC3T3 osteoblast-like cells	SLA	Cells cultured on titanium discs alone or with EMD or DMD at 100 μg/mL, 1, 10, and 30 mg/mL	Adhesion: 2, 4, and 8 h Proliferation, viability: 24, 48, and 72 h Cell migration (assessed with scratch wound healing model): 24 h Gene expression for osteoblast differentiation and bone formation (Runx2, COL1A2, ALP, BSP): 24 h	Increased osteoblasts proliferation and viability both with EMD and DMD; increased gene expression with EMD or DMD concentrations ≥10 mg/mL

ALP: Alkaline phosphatase; Ang-2: Angopoietin-2; BSP: Bone sialoprotein; COL1A1: Collagen 1a1; COL1A2: Collagen 1a2; DMD: Dentin matrix derivative; EMD: Enamel matrix derivative; EPCR: Endothelial protein C receptor; FN1: Fibronectin-1; HUVECs: Human umbilical vein endothelial cells; ICAM-1: Intercellular adhesion molecule-1; LM: Light microscopy; OPG: Osteoprotegerin; RANKL: Receptor activator of nuclear factor κB ligand; SEM: Scanning electron microscopy; SLA: Sand-blasted, large grit, acid-etched; VEGF-A: Vascular endothelial growth factor-A; vWF: Von Willebrand Factor.

**Table 2:** In vivo studies on the effect of EMD on implants in animal models.

Authors	Year	Animal Model	Design of the Study	Methods	Results
Shimizu-Ishiura et al. [20]	2002	Rats	EMD vs. control at implant placement	Mini-implants (3.5 × Ø 1.6 mm) placement in rat femur with EMD or PGA (control); dissection at 4, 7, 14, and 30 days; analysis by LM, SEM, immunohistochemistry, and backscattered electron image analysis	EMD enhanced trabecular bone formation around implants
Casati et al. [21]	2002	Mongrel dogs	EMD vs. EMD + GBR vs. control for peri-implant bone dehiscence treatment	Mandibular teeth extraction in six dogs; at 3 months: 4 osteotomies with dehiscence-type defects, implant placement, application of EMD, EMD + GBR, or nothing; at 5 months: Four additional defects; at 6 months: Animal sacrifice	No statistically significant difference in BIC; new bone area was significantly higher in EMD + GBR vs. control samples
Franke Stenport & Johansson [22]	2003	New Zealand white rabbits	EMD vs. control at implant placement	Placement of 36 implants in six rabbits, with EMD or PGA (control); dissection at 6 weeks; analysis by RFA, RTQ, histomorphometric analysis	No statistically significant difference
Craig et al. [23]	2006	Minipig	PDL or GCT cells alone or +EMD for peri-implant bone dehiscence treatment	Mandibular and maxillary teeth extraction in one minipig 6 and 3 weeks before implant placement, respectively; implant placement with PDL or GCT cells alone or +EMD, coverage with a bioabsorbable membrane; animal sacrifice at 8 weeks; histomorphometric analysis	GCT cells led to formation of fibrous connective tissue; PDL cells alone led to good BIC but with strands of epithelium; PDL + EMD led to good BIC contact without epithelium
Hurzeler et al. [24]	2010	Beagle dog	EMD at implant placement with socket shield technique	Hemisection of mandibular premolars in one beagle dog, retention of buccal radicular fragment, placement of four implants +EMD, with or without contact with radicular fragment; animal sacrifice at 4 months; histological analysis, backscatter scanning electron microscopy	Osseointegration of all implants, with new cementum on implants placed in direct contact with radicular fragments
Birang et al. [25]	2012	Iranian dogs	EMD vs. control at implant placement	Placement of 12 implants in three dogs, alone or +EMD; animal sacrifice at 2, 4, and 6 weeks; immunohistochemical and histomorphometric analyses	Significantly higher proliferation of osteoblasts, no statistical difference in bone formation
Wen et al. [26]	2015	Rabbit	EMD + DBBM, BCPT1 or BCPT2 for vertical bone regeneration	Partial insertion of 30 implants + three different materials: DBBM, BCPT1 (granule size 500–1000 µm) or BCPT2 (granule size 250–100 µm) in 15 rabbits: Each animal received one implant with one biomaterial alone and one with the same biomaterial +EMD; animal sacrifice at 10 weeks; histomorphometric analysis	Higher bone density with EMD adjunct when DBBM and BCPT2 were used; no statistically significant differences in BIC and bone height
Lim et al. [27]	2016	Mongrel dogs	EMD alone, BCP alone, EMD + BCP vs. control for peri-implant bone dehiscence treatment	Osteotomies with circumferential defects in five dogs, implant placement + biomaterials, scheduled in order to have a 2- and 8-week follow-up for each dog; histometric analysis	No statistically significant differences in bone formation, BIC, and defect fill
Ikawa et al. [28]	2019	Beagle dogs	NBB alone, NBB + EMD vs. control for buccal bone dehiscence treatment	Teeth extraction 3 months before implant placement in six dogs; osteotomies with dehiscence-type defects, placement of three implants per dog and application of NBB alone, NBB + EMD or nothing, covered with collagen membrane; histomorphometric analysis after 3 months	Higher new bone formation and BIC in test groups vs. control group; first BIC was significantly more coronal with EMD

BCPT1: Macro-structuring BiPhasic HA/ß TCP particulate bone graft substitute; BCPT2: Micro-structuring BiPhasic HA/ß TCP particulate bone graft substitute; BIC: Bone to implant contact; DBBM: Deproteinized bovine bone mineral; EMD: Enamel matrix derivative; GBR: Guided bone regeneration; GCT: Gingival connective tissue; LM: Light microscopy; NBB: Natural bovine bone; PDL: Periodontal ligament; PGA: Propylene glycol alginate; RFA: Resonance frequency analysis; RTQ: Removal torque measurement; SEM: Scanning electron microscopy.

**Table 3:** Studies evaluating the use of EMD at implant sites in humans.

Authors	Year	Study Type	Population	Methods	Results
Cangini and Cornelini [29]	2005	Clinical report	32 implants in 32 patients, mean age 45 years (age range 21–60 years), 10 smokers	Implant placement immediately after tooth extraction, filling of the remaining bone defect with EMD or bioresorbable collagen membrane; final prosthetic restoration at 6 months; clinical evaluation at 12 months	Membrane led to lower PD and better position of the soft tissue margin around the implant shoulder
Froum et al. [30]	2012	Case series	51 implants in 38 patiens, age range 29–81 years	Treatment of peri-implantitis through surface decontamination, + EMD, + PDGF + anorganic bovine bone or mineralized freeze-dried bone, + coverage with a collagen membrane or a subepithelial connective tissue graft; follow-up at 3 to 7.5 years	Mean PD reduction of 5.4 and 5.1 mm in mesial/distal defects and buccal/lingual defects, respectively; mean bone level gain of 3.75 and 3.0 mm
Froum et al. [31]	2015	Case series	170 implants in 100 patients	As reported by Froum et al. 2012; follow-up at 2–10 years. Surgical procedures were repeated twice in 18 implants and three times in 10 implants	Survival rate 98.8%, mean PD reduction 5.10 mm, mean bone level gain 1.77 mm, mean soft tissue marginal gain 0.52 mm
Guimaraes et al. [32]	2015	Clinical study	Five patients, without periodontal disease, diabetes, not smoking	Split mouth design: Placement of at least one implant in each side of the maxilla, one side with EMD adjunct; second-stage surgery at 14 days with biopsies; histologic and immunohistochemical analysis	Increased number of blood vessels in EMD-treated sites
Faramarzi et al. [33]	2016	Double-blind RCT	64 patients, not smoking	Non-surgical treatment of peri-implant mucositis alone, +EMD or +MSM; clinical evaluation and microbial analysis of peri-implant crevicular fluid at 2 weeks and 3 months	Significant decrease in *P. gingivalis* levels, PD and BOP for both EMD and MSM groups
Isehed et al. [34]	2016	Double-blind RCT	26 patients	Surgical treatment of peri-implantitis alone or +EMD; clinical, radiographic, and microbiologic evaluation of PICF at 3, 6, and 12 months	EMD led to significantly higher marginal bone levels at 12 months and was associated to a Gram+/aerobic microbial flora
Kashefimehr et al. [35]	2017	Double-blind RCT	46 patients, not smoking	Non-surgical treatment of peri-implant mucositis alone or +EMD, clinical and microbiologic evaluation of PICF at 3 months	Significant improvements in BOP and PD, decrease in IL-6 and IL-7 levels in PICF
Isehed et al. [36]	2018	RCT	25 patients, 18 screened at 3 years, 14 at 5 years	Surgical treatment of peri-implantitis alone or +EMD; clinical and radiographic evaluation at 3 and 5 years	EMD was positively associated with implant survival at 5-year follow-up
Esberg et al. [37]	2019	RCT	25 patients, 25 implants	Surgical treatment of peri-implantitis alone or +EMD; evaluation of PICF proteomic profile at 3, 6, and 12 months; analysis of its correlation with EMD adjunct, PD, implant survival up to 5 years	Two main clusters were identified, with different proteomic profiles. One was related to implant loss and higher protein concentration and diversity; the other one was related to implant survival at 5 years and EMD treatment
Cardaropoli et al. [38]	2019	Clinical study	20 patients	Placement of immediate post-extraction anterior maxillary single-tooth implant + xenograft, application of EMD and placement of immediately loaded screwed restoration; evaluation of soft tissue contour at 12 months	No difference in soft tissue contour between pretreatment and 12-month follow-up
Cardaropoli et al. [39]	2019	Clinical study	20 patients	As reported by Cardaropoli et al. 2019; definitive ceramic crown at 3 months; radiographic and CBCT evaluation at 12 months	No difference in marginal bone levels and horizontal width of bone crest between pretreatment and 12-month follow-up

BOP: Bleeding on probing; EMD: Enamel matrix derivative; F: Female; M: Male; MSM: Micro-spherical minocycline; PD: Probing depth; PICF: Peri-implant crevicular fluid; RCT: Randomized clinical trial.

## Data Availability

The data presented in this study is contained within the article.

## Appendix A

Search String for Medline via Ovid

1. (‘amelogenin’ or ‘amelogenin’ or ‘amelogenins’ or ‘dental enamel proteins’ or ‘enamel proteins, dental’ or ‘proteins, dental enamel’). mp. or exp ‘Amelogenin’/use medall or exp ‘Dental Enamel Proteins’/use medal
2. ‘emdogain’. mp.
3. ‘enamel matrix derivative’. mp.
4. 1 or 2 or 3
5. limit 4 to ‘humans only (removes records about animals)’
6. (‘dental implant’ or ‘dental implant’ or ‘dental implants’ or ‘dental prostheses, surgical’ or ‘dental prosthesis, surgical’ or ‘implant, dental’ or ‘implants, dental’ or ‘prostheses, surgical dental’ or ‘prosthesis, surgical dental’ or ‘surgical dental prostheses’ or ‘surgical dental prosthesis’). mp. or exp ‘Dental Implants’/use medall
7. limit 6 to ‘humans only (removes records about animals)’
8. 5 and 7

Embase

(‘enamel matrix proteins’/exp OR ‘enamel matrix proteins’ OR ‘enamel matrix protein’/exp OR ‘enamel matrix protein’ OR ‘enamel matrix derivative’/exp OR ‘enamel matrix derivative’ OR ‘amelogenin’/exp OR ‘amelogenin’ OR ‘emdogain’/exp OR ‘emdogain’) AND (‘tooth implant’/exp OR ‘tooth implant’ OR ‘dental implant’ OR ‘dental implants’) AND (humans)/lim
